# Benchmarking Hospital Practices and Policies on Intrahospital Neurocritical Care Transport: The Safe-Neuro-Transport Study

**DOI:** 10.3390/jcm12093183

**Published:** 2023-04-28

**Authors:** Kapil G. Zirpe, Bhunyawee Alunpipatthanachai, Nassim Matin, Bernice G. Gulek, Patricia A. Blissitt, Katherine Palmieri, Kathryn Rosenblatt, Umeshkumar Athiraman, Suneeta Gollapudy, Marie Angele Theard, Sarah Wahlster, Monica S. Vavilala, Abhijit V. Lele, Safe-Neuro-Transport Collaborators

**Affiliations:** 1 Neurotrauma Unit, Ruby Hall Clinic, Pune 411040, India; kapilzirpe@gmail.com; 2 Bhunyawee Alunpipatthanachai, Bhumibol Adulyadej Hospital, Bangkok 10220, Thailand; bhunyav@hotmail.com; 3 Neurocritical Care Service, Department of Neurology, Harborview Medical Center, University of Washington, Seattle, WA 98104, USA; nmatin@uw.edu (N.M.); wahlster@uw.edu (S.W.); 4 Harborview Medical Center, University of Washington School of Nursing, Seattle, WA 98104, USA; 5 Department of Anesthesiology, University of Kansas Health System, Kansas City, KS 66160, USA; palmiek@kumc.edu; 6 Department of Anesthesiology, Johns Hopkins Medical Institutions, Baltimore, MD 21287, USA; krosenb3@jhmi.edu; 7 Department of Anesthesiology, Washington University, St. Louis, MO 63130, USA; 8 Medical College of Wisconsin, Milwaukee, WI 53226, USA; gsuneeta@mcw.edu; 9 Department of Anesthesiology and Pain Medicine, Harborview Injury Prevention and Research Center, Harborview Medical Center, University of Washington, Seattle, WA 98122, USA; mthear@uw.edu (M.A.T.); vavilala@uw.edu (M.S.V.); 10 Neurocritical Care Service, Department of Anesthesiology and Pain Medicine, Harborview Injury Prevention and Research Center, Harborview Medical Center, University of Washington, Seattle, WA 98122, USA

**Keywords:** critical care transport, intra-hospital transport, complications, policies, standard operating procedures, adverse events, quality, safety, neurocritical care, alignment, adherence

## Abstract

An electronic survey was administered to multidisciplinary neurocritical care providers at 365 hospitals in 32 countries to describe intrahospital transport (IHT) practices of neurocritically ill patients at their institutions. The reported IHT practices were stratified by World Bank country income level. Variability between high-income (HIC) and low/middle-income (LMIC) groups, as well as variability between hospitals within countries, were expressed as counts/percentages and intracluster correlation coefficients (ICCs) with a 95% confidence interval (CI). A total of 246 hospitals (67% response rate; *n* = 103, 42% HIC and *n* = 143, 58% LMIC) participated. LMIC hospitals were less likely to report a portable CT scanner (RR 0.39, 95% CI [0.23; 0.67]), more likely to report a pre-IHT checklist (RR 2.18, 95% CI [1.53; 3.11]), and more likely to report that intensive care unit (ICU) physicians routinely participated in IHTs (RR 1.33, 95% CI [1.02; 1.72]). Between- and across-country variation were highest for pre-IHT external ventricular drain clamp tolerance (reported by 40% of the hospitals, ICC 0.22, 95% CI 0.00–0.46) and end-tidal carbon dioxide monitoring during IHT (reported by 29% of the hospitals, ICC 0.46, 95% CI 0.07–0.71). Brain tissue oxygenation monitoring during IHT was reported by only 9% of the participating hospitals. An IHT standard operating procedure (SOP)/hospital policy (HP) was reported by 37% (*n* = 90); HIC: 43% (*n*= 44) vs. LMIC: 32% (*n* = 46), *p* = 0.56. Amongst the IHT SOP/HPs reviewed (*n* = 13), 90% did not address the continuation of hemodynamic and neurophysiological monitoring during IHT. In conclusion, the development of a neurocritical-care-specific IHT SOP/HP as well as the alignment of practices related to the IHT of neurocritically ill patients are urgent unmet needs. Inconsistent standards related to neurophysiological monitoring during IHT warrant in-depth scrutiny across hospitals and suggest a need for international guidelines for neurocritical care IHT.

## 1. Introduction

Patients admitted to an intensive care unit (ICU) with neurological/neurosurgical emergencies are frequently transported to and from the ICU [1], as they may require urgent imaging studies, neurosurgical procedures, and other diagnostic as well as therapeutic interventions. Intrahospital transports can be performed under routine, urgent, or emergent conditions, as well as in patients with varied risks for IHT-related adverse events (AEs) [2,3,4]; however, IHTs may be associated with adverse events [1,5,6,7,8,9,10,11,12]. Specific concerns for neurocritically ill patients with IHTs include the precipitation of secondary brain injuries related to reduced brain tissue oxygenation, cerebral perfusion pressure reductions, intracranial pressure elevations, and inadequate cerebrospinal fluid drainage. Critical care transports are a source of stress and increased workload [13,14].

In 1993, the Society of Critical Care Medicine (SCCM) first put forth the best practices for the safe conduct of critical care intrahospital transports (IHTs) [15]. This was followed in 2004 by the American College of Critical Care Medicine (ACCM) guidelines [16]; however, these guidelines do not include any neurocritical-care-specific elements. The European Society of Intensive Care Medicine (ESICM) [17] published recommendations for the intra-hospital transport of severely head-injured patients in 1999 and focused on five areas: (1) pre-transport stabilization, (2) pre-transport coordination and communication, (3) accompanying staff, (4) transport and monitoring equipment, and (5) documentation. Specifically, the ESICM recommended the pre-transport stabilization of the patient with the fohospital transport of the severely llowing target end points in adults: mean arterial blood pressure >90 mmHg, systolic arterial blood pressure >120 mmHg, intracranial pressure <20 mmHg, and cerebral perfusion pressure >70 mmHg. In addition, recommendations also include documentation of the patient’s neurological status before, during, and after transport. A patient at risk of acute neurological deterioration should be accompanied by a physician and at least two other individuals during IHT.

While few would argue that critical care IHTs’ best practices should focus on safe transport, it is unknown whether prevalent institutional IHT practices and policies are aligned with guidelines and whether neurocritical care elements are taken into consideration. We conducted a study to describe prevalent IHT practices worldwide and compare institutional policies with SCCM/ACCM/ESICM guidelines/recommendations. This study aimed to examine the alignment of hospitals’ standard operating procedures (SOPs) and policies (HPs) related to IHTs with available recommendations and to explore variations in SOPs and HPs by World Bank country income level. 

## 2. Materials and Methods

### 2.1. Institutional Review Board Approval

This study was approved on 21 March 2022 by the University of Washington Institutional Review Board (STUDY00014283). The survey invitation informed participants that participation was voluntary, without any financial incentives. Informed consent was obtained from the participants and responses were de-identified at the institutional level. 

### 2.2. Survey of Hospital IHT Practices

An electronic survey was conducted between 1 March 2022 and 30 June 2022. Study sites were identified from a list serve of neuroanesthesiology and neurocritical care fellowship programs advertised on the Society of Neuroscience in Anesthesiology and Critical Care [18] and the United Council for Neurologic Subspecialties websites [19]. In addition, we also included collaborators with a track record in neurocritical care research [20]. We collected only one response per hospital and aimed to target multidisciplinary healthcare providers who routinely cared for neurocritically ill patients.

The survey (Supplementary Digital Content #1) was designed using the Research Electronic Data Capture (REDCap) system hosted by the University of Washington’s Institute of Translational Health Sciences REDCap electronic data capture tools [21] and administered in English as well as Spanish. A total of 104 questions were designed to examine prevalent IHT practices, with questions based on the SCCM [15], ACCM [16], and ESICM guidelines. The survey covered the following areas: clinical setting, the presence of IHT-SOP/HP, the personnel involved in IHT, pre-IHT assessments of patients, contraindications to IHT, equipment as well as monitoring standards, and adverse events associated with critical care IHT. In addition, neurological-critical-care-specific areas included the presence of portable computerized tomography (CT) and the defined allocation of portable CT, intracranial pressure monitoring, and reports of neurological as well as non-neurological adverse events (AEs) associated with IHTs. 

Survey responders were de-identified for 18 Health Insurance Portability and Accountability Act identifiers. Neurocritical care experts conducted internal and external peer reviews of the survey for content and errors, and pilot responses were not included in the final analysis. A link to the survey was emailed to potential respondents, followed by five reminders that were sent one week apart. The data presented were de-identified at the hospital level. 

### 2.3. Review of Intrahospital Transport Standard Operating Procedures/Hospital Policies

N.M. conducted a comprehensive review of the SOPs/HPs shared by the study collaborators: To assess adherence to the recommendations for safe intrahospital transfer proposed in the published SCCM/ACCM/ESICM guidelines, we compiled 58 recommendations and categorized them as follows: scope of the policy, risk stratification criteria, pre-transport assessment/checklist, transport personnel, transport equipment as well as monitoring, continuation of ICU care, and references cited. 

### 2.4. Statistical Analysis

Descriptive statistics informed cohort characteristics. Responses are reported as counts and percentages. Variations in reported practices by World Bank country income level and between hospitals located by country are expressed using intracluster correlation coefficients (ICCs) and 95% confidence intervals (CIs). The intracluster correlation coefficient (ICC) for each continuous variable is calculated by directly estimating the between-cluster (σ2c) and within-cluster (σ2w) variances in a mixed model that treated clusters as random effects [22]. Since the random intercept model did not estimate ICCs from sample data, bootstrap estimates (distribution) from 1000 bootstrap samples are reported [23]. Differences between HMIC and LMIC hospitals are analyzed using chi-square tests, the calculation of relative risk ratios (RRs) for LMIC responses, and the calculation of 95% confidence intervals (CIs). A Bonferroni-corrected *p*-value of < 0.05 indicates statistical significance. STATA 15 [24]/RStudio 1.554 [25] was used for the statistical analysis, and Prism GraphPad [26] was used for creating forest plots.

## 3. Results

### 3.1. Participating Institutions 

**Figure 1:**
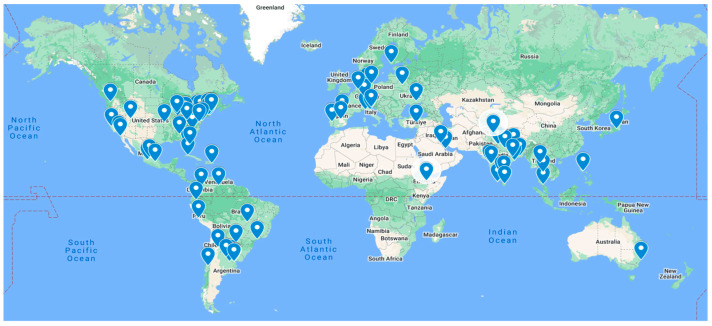
World Map Showing Safe-Neuro-Transport Study Participating Sites.

Of the 365 hospitals that were identified and contacted, responses were received from 246 hospitals (response rate of 67.4%), representing 32 countries (Figure 1), with 41.9% of sites from HICs (*n* = 103) and 58.1% from LMICs.

Participant and hospital characteristics are detailed in Table 1. Respondents were from teaching hospitals (*n* = 227, 92.3%), comprehensive stroke centers (*n* = 153, 62.2%), and level I trauma centers (*n* = 94, 38.2%), and reported the presence of more than one intensive care unit (*n* = 197, 80.1%) at their hospital.

### 3.2. Clinical Setting of Participating Hospitals Where Critical Care IHT Is Conducted 

Personnel involved in the routine transport of critically ill patients included nurses (*n* = 157, 69%), respiratory therapists (*n* = 102, 45%), physicians (*n* = 128, 52%), advanced practice providers (*n* = 53, 23%), and members of a dedicated transport team (*n* = 76, 33%). Overall, 119 (48.8%) reported a minimum of two people required for all transports, and 46 (18.7%) reported no minimum number. Overall, 115 (46.7%) said that training/proficiency/clinical competency or IHT personnel is benchmarked by institutional leadership, and 160 (65%) reported that supervision is provided to inexperienced trainees/house staff/nurses involved in IHT. Regarding clinical competency for IHT, responses were mixed; the majority (*n* = 188, 76%) mentioned the involvement of either an ICU nurse or an ICU physician, some specifically said advanced cardiac life support (*n* = 11, 4%) or basic life support (*n* = 4, 2%) certification, and some (*n* = 2, 1%) mention local institutional training required for IHT. In contrast, others (*n* = 2, 1%) mentioned no specific IHT-related training requirement, and one mentioned “whoever is available for transport”. There was one mention of conducting mock IHT drills. 

#### Portable CT Scan

Forty-eight (19.5%, HICs: 31, 64.6%; LMICs: 17, 35.4%) hospitals reported the availability of a portable CT scanner, with LMICs less likely to report the presence of a portable CT at their institution (RR 0.39, 95% CI [0.23; 0.67], *p* = 0.0005). Of these, 48 hospitals (*n* = 26, 54%) reported established criteria specifying how patients are selected to receive portable CT. Amongst those who reported defined criteria, hemodynamic or neurological instability (e.g., rapid decline in the level of consciousness) was cited as the most common reason (*n* = 17/26, 65.3%) for using a portable CT. A dedicated IHT team was less likely to be reported by LMIC hospitals (RR 0.65, 95% CI [0.45; 0.94], *p* = 0.03).

### 3.3. Preparation/Assessment of a Patient before the Initiation of IHT

Pre-IHT preparation included the identification of qualified personnel that accompany the patient (*n* = 147, 65%), identifying risks posed to the patient during transport (*n* = 130, 57%), equipment required to accompany the patient (*n* = 155, 68%), physician–physician or nurse–nurse communication regarding a patient’s condition and treatment during transport (*n* = 126, 56%), confirmation that the receiving area is ready for the patient to be transported (*n* = 147, 65%), notification of ancillary services as to the timing of the transport and the equipment and/or support that they will need to provide (*n*= 119, 52%), notification of physicians to be aware of the impending IHT (*n* = 102, 45%), documentation of IHT indication in the medical records (*n* = 89, 39%), documentation of the patient’s neurological status before IHT (*n* = 91, 40%), and the risk stratification of IHT into low-risk vs. moderate-risk vs. high-risk (*n* = 90, 40%). Variations between HICs and LMICs regarding the preparation/assessment of patients before IHT are shown in Table 2. Between- and across-country variation in pre-IHT assessment/preparation was highest for external ventricular drain clamp tolerance testing before initiating IHT (ICC 0.22, 95% CI 0.00–0.46). 

### 3.4. Equipment and Monitoring during Critical Care

Overall, the equipment/monitoring reported during critical care IHT was as follows: pulse oximetry (94%), oxygen source (90%), resuscitation bag (81%), standard resuscitation drugs (70%), transport ventilator (69%), stethoscope (69%), cardiac telemonitoring (67%), and endotracheal tube as well as airway (60%). Differences between HIC and LMIC hospital practices regarding equipment and monitoring standards during critical care IHT are shown in Table 3. LMIC and HIC hospitals reported a lack of equipment: elevator key (*n* = 57, 25%) and transport trolley with an oxygen source, as well as a place for monitoring equipment (*n* = 34, 15%). Between- and across-country variation were highest for end-tidal carbon dioxide monitoring (ICC 0.46, 95% CI 0.07–0.71).

### 3.5. Reported Neurological and Non-Neurological Adverse Events Occurring during Intrahospital Transport 

The reported occurrence of system-wise adverse events (AEs) was as follows: 

Neurological AEs: These included agitation and restlessness (55%), a reduction in cerebral perfusion pressure (18%), elevated intracranial pressure (38%), cerebral herniation (15%), a decrease in brain tissue oxygenation (15%), the dislodgement of an external ventricular drain (26%), the dislodgement of a lumbar drain (18%), the overdrainage of cerebrospinal fluid due to the disconnection of an external ventricular drain (17%), and the overdrainage of cerebrospinal fluid due to the disconnection of a lumbar drain (10%). 

LMIC hospitals were less likely to report the occurrences of the following neurological AEs: a reduction in cerebral perfusion pressure (RR 0.34, 95% CI 0.19–0.60), a reduction in intracranial pressure (RR 0.43, 95% CI 0.31–0.61), cerebral herniation (RR 0.37, 95% CI 0.20–0.69), agitation and restlessness (RR 0.69, 95% CI 0.55–0.88), and the overdrainage of cerebrospinal fluid due to disconnection of an external ventricular drain (RR 0.42, 95% CI 0.23–0.74).

Specifically related to neurological AEs, we observed that hospitals with the presence of a portable scanner were more likely (25% vs. 13%, RR 1.9, 95% CI 1,04–3.49) to report the occurrence of cerebral herniation AEs during IHT compared to hospitals that reported the absence of a portable scanner; however, the occurrence of other neurological AEs was not significantly different between the two groups.

The most common reports of other systemic AEs occurring during IHT were included—cardiovascular: hypotension (52%); pulmonary: equipment-related hypoxia (57%) and disconnection from the mechanical ventilator (43%); and gastrointestinal: vomiting (31%). 

**Figure 2:**
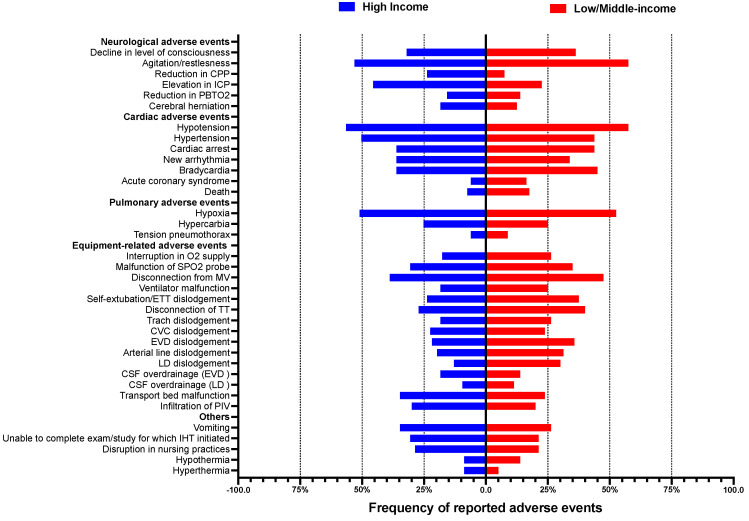
Reports of critical care intrahospital transport-related adverse events occurring during IHT in high-Income and low–middle-income countries.

Differences between the reports of AEs occurring during IHT, stratified by country income level, are presented in Figure 2.

### 3.6. Presence of IHT Standard Operating Procedures/Hospital Policies 

An IHT standard operating procedure (SOP)/hospital policy (HP) was reported by 90 (36.6%) hospitals: 44 (42.7%) from HICs and 46 (32.2%) from LMICs. Clinicians involved in drafting IHT-SOP/HP varied—ICU physician leadership: *n* = 63 (72.4%), ICU nursing leadership: *n* = 53 (61%), anesthesiology leadership: *n* = 24 (28%), emergency departmental leadership: *n* = 27 (31%), hospital nursing leadership: *n* = 38 (44%), and a critical care committee: *n* = 31, (36%). Most hospitals (80%) with IHT-SOP/HP reported that their policy was updated/revised within the last five years. Policies were either a single policy/institution that addressed adults and children, all ICUs with unit-specific sections: *n* = 31 (14%), or there was more than one policy per intensive care unit or for adults and children: *n* = 34 (15%). Many participants (*n* = 162, 71%) reported not knowing the details of their institution’s SOP/HPs. 

### 3.7. Review of IHT SOPs/HPs 

Of the 90 hospitals reporting an IHT SOP/HP, 13 shared their hospital policies for review. The common reason for the low number of SOPs/HPs shared was the presence of rigorous hospital regulations of not sharing proprietary, confidential documents, as disclosed by our participants. The majority of the policies reviewed (*n* = 9, 69%) were from the United States; other policies were from hospitals located in India (*n* = 1), Australia (*n* = 1), Thailand (*n* = 1), and the United Arab Emirates (*n* = 1). Of the policies, 4 (31%) were last updated in 2020 and 9 (69%) were updated in 2021. 

We did not identify any hospital policy with 100% adherence to all components of the standard recommendations. We observed that 100% of the policies included some IHT checklist, 90% did not specify contraindications to IHT, 59% mentioned some IHT equipment, 40% mentioned a pre-transport specific checklist, 27.3% mentioned mandatory qualifications of IHT personnel, 25% included IHT-related communication, 20% included risk stratification and identification of physiological red flags for transport, 20% included documentation of peri-IHT examinations as well as events, and no policy mentioned audits in addition to quality assurance processes. 

Specific to neurocritical care, we found only one policy that addressed details concerning IHT with indwelling EVD, such as clamp tolerance or risk of intracranial pressure elevation. In contrast, only two policies included guidance about intracranial pressure monitoring, and no policies mentioned brain tissue oxygenation. The continuation of hemodynamic and neurophysiological monitoring during IHT was missing in 90% of the policies with regard to temperature, end-tidal carbon dioxide, cerebral perfusion pressure, intracranial pressure, and brain oxygenation. Similarly, documentation of the Glasgow Coma Scale score, intracranial pressure, cerebral perfusion pressure, and other neurological data points in electronic medical records was not mentioned in 80% of the policies. Of the policies, 59% did not mention any post-transport communication, 38% mentioned a hand-off, and only 3% mentioned maintaining a record of vital signs. One policy mentioned maintaining the dignity and safety of all patients and that, for minors/unaccompanied females, one female adult must accompany them. 

Regarding the references listed in IHT policies, 3/23 (23%) policies did not mention any references. Only 3 (23%) referenced SCCM guidelines, and only 3 (23%) referenced ACCM guidelines. 

### 3.8. Differences between Country Income Levels and Intrahospital Transport Practices of Critically Ill Patients 

**Figure 3:**
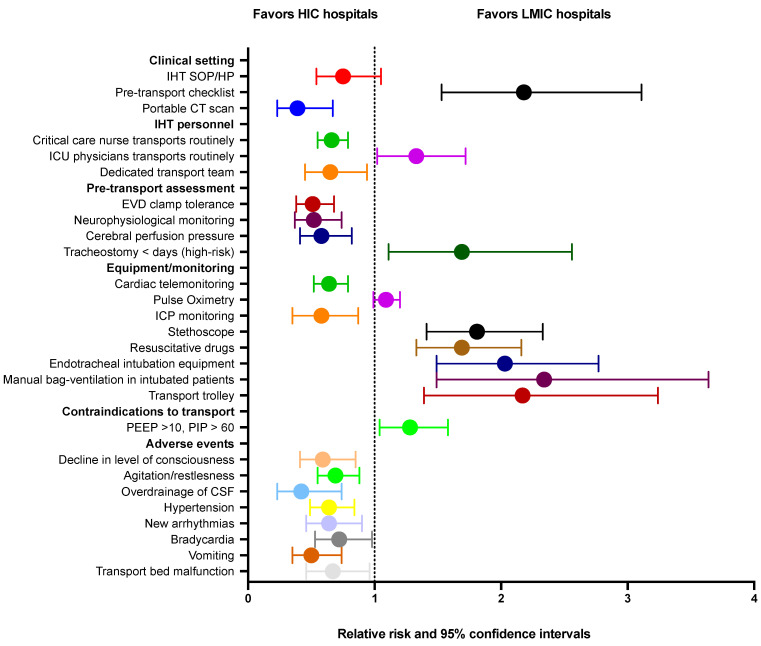
Association between country income level and intrahospital transport of critically ill patient practices. Abbreviations: HIC: high-income country; LMIC: low/middle-income country; IHT: intrahospital transport; SOP: standard operating procedure; HP: hospital policy; ICU: intensive care unit; EVD: external ventricular drain; ICP: intracranial pressure; PEEP: positive end-expiratory pressure; PIP: peak inspiratory pressure; and CSF: cerebrospinal fluid.

The differences between clinical settings, pre-IHT assessments, contraindications to transport, equipment/monitoring standards, and reported adverse events from HIC as well as LMIC hospitals are shown in Figure 3.

As shown in Figure 3, LMIC hospitals were less likely to report the availability of a portable CT scanner (RR 0.39, 95% CI [0.23; 0.67]), critical care nurses routinely involved in IHT (RR 0.66, 95% CI [0.55; 0.79]), and were less likely to report adverse events, such as a decline in the level of consciousness (RR 0.59, 95% CI [0.41; 0.85], agitation/restlessness (RR 0.69, 95% CI [0.55; 0.88]), the overdrainage of cerebrospinal fluid ((RR 0.42, 95% CI [0.23; 0.74]), cardiac arrhythmias (RR 0.64, 95% CI [0.46; 0.90]), bradycardia (RR 0.72, 95% CI [0.53; 0.98]), hypertension (RR 0.64, 95% CI [0.49; 0.84], vomiting (RR 0.50, 95% CI [0.35; 0.74]), and transport bed malfunction (RR 0.67, 95% CI [0.46; 0.96]). As shown in Figure 3, LMIC hospitals were more likely to report the presence of a pre-IHT checklist (RR 2.18, 95% CI [1.53; 3.11]), ICU physicians routinely performing IHT (RR 1.33, 95% CI [1.02; 1.72]), not undertaking routine IHT in patients with a tracheotomy <7 days old (RR 1.69, 95% CI [1.11; 2.58]), or not transporting patients with PEEP > 10 or those with peak airway pressure >60 (RR 1.28, 95% CI [1.04; 1.58]), carrying a stethoscope during IHT (RR 1.81, 95% CI [1.41; 2.33]), carrying resuscitative drugs (RR 1.69, 95% CI [1.33; 2.16]), carrying equipment for endotracheal intubation (RR 2.02, 95% CI [1.49; 2.77]), manually hand-ventilating intubated patients (RR 2.34, 95% CI [1.49; 3.64]), and carrying a transport trolley (RR 2.17, 95% CI [1.39; 3.24]). 

## 4. Discussion

An international-survey-based study examined prevalent practices regarding neurocritical care IHT amongst hospitals from 32 countries. The main findings of this study are as follows: (1) existing practices related to neurocritical IHT SOPs/HPs lack neurocritical-specific elements such as intracranial pressure, cerebral perfusion pressure as well as brain tissue oxygen monitoring and management, and management of cerebrospinal fluid diversion devices, such as external ventricular and lumbar drains; (2) only half of the hospitals reported an IHT SOP/HP; (3) there are differences between pre-IHT preparation, the assessment of patients, as well as equipment and monitoring standards during IHTs involving critically ill patients between HIC and LMIC hospitals; (4) neurological as well as non-neurological adverse events are commonly reported during IHT, in addition to the fact that the reporting of the AEs may differ by country income level; and (5) hospital IHT policies are not comprehensive and not concordant with published guidelines as well as recommendations.

Our study finds a lack of incorporation of neurocritical-care-specific elements into hospital IHT SOP/HPs and reported practices. Prior research by Klefmann et al. in 2016 [27] reported the rate of complications during the intrahospital transport of critically ill patients with severe brain diseases, with a significant increase in intracranial pressure during transport and CT scans. In one-fifth of all patients, it was reported that additional therapy was necessary. We agree with their point of view that the transport of critically ill patients should only be performed by trained staff and under the monitoring of intracranial pressure as well as cerebral perfusion pressure. Chaikitaasilpa et al. [1] reported an increased risk of intracranial pressure elevation during neurocritical IHTs in a patient with indwelling EVDs, in a clinical setting where the prevalent practice is routinely clamping their EVD during IHTs. Unfortunately, neither the SCCM, ACCM, nor ESICM guidelines recommend intracranial pressure as well as cerebral perfusion pressure monitoring and provide details regarding managing cerebrospinal fluid diversion devices commonly placed in neurocritical care units. It could also be that the lack of the implementation of published recommendations in clinical practice suggests that neurocritical IHTs perhaps are not given the high priority that they deserve amongst neurocritical care units; this may be because the guidelines do not mention it. Neurocritical care providers should not take IHTs for granted but spend some time reviewing the need for IHT. We agree with the ESICM statement that “the decision to move such a patient must be based on the assessment of the potential benefits of the diagnostic test, the procedural intervention, or the higher level of care (better technology and/or specialists) weighed against the potential hazards of transport. If the action is unlikely to alter the management or outcome of the patient positively, then the need to move the critically ill patient must be questioned. Alternative bedside tests and procedures must always be considered.” 

Some hospitals (HICs more than LMICs) report the presence of a portable CT scanner; however, our study found no differences in reported neurological AEs except for cerebral herniation, reported largely from hospitals with portable CT scanners. It could be possible that portable CT is underutilized and that patients are routinely, urgently, and emergently transported to other areas of hospitals for their CTs due to a lack of availability of personnel to manage a portable CT scan or a time lag in making the equipment ready to start the scanning procedure. It could also be due to (and we did not test this in our study) higher-severity illness patients being admitted to those hospitals reporting a higher rate of cerebral herniation. Peace et al. reported that portable head CT scans do not have a detectable effect on a critically ill patient’s intracranial pressure, cerebral perfusion pressure, or brain tissue oxygenation [28]. LaRovere reported that two-thirds of CT scans obtained in the pediatric intensive care units were portable because of patients’ intensity of therapy and illness severity, implying a strategy that prioritizes portable scans as opposed to IHTs [29]. As with portable CT scanners, portable MRI machines are now available, and we are just learning how to use them in the neurocritical care unit [30]. 

Our study finds that only half of the hospitals worldwide have an IHT SOP/HP. Considering that critical care IHTs may be associated with neurological and non-neurological AEs, the lack of a reference document with which to guide neurocritical care clinicians to perform safe IHT is alarming. Similarly, it is worth discussing that the mere presence of a policy is insufficient to assure quality and safety during critical care IHT. The review of IHT policies (albeit a few were available for review) suggests that a checklist/protocol may exist to allow consistency in IHT [13,31,32,33]. The other vital areas, such as transport equipment, risk stratification, the identification of physiological red flags for transport, peri-transport communications, documentation of peri-transport examination and events, performing recurrent audits, and conducting quality assurance meetings, may not be as evolved as may be expected. 

The observed differences between pre-IHT preparation as well as the assessment of patients, equipment, and monitoring standards during IHTs involving critically ill patients between HIC and LMIC hospitals are not surprising, given the variability in available equipment and resources at individual hospitals. Specific to neurocritical care, the inconsistent continuation of the neurophysiological monitoring of IHT is concerning. Through our study, we propose increasing the awareness of the risk of complications associated with external ventricular drains, the performance of a clamp trial before initiating IHT, monitoring intracranial pressure during IHT, and suggesting vigilance to maintaining cerebral perfusion pressure as well as prevent inadvertent hypocapnia/hypercapnia. 

A broader review of the IHT process at individual hospitals, followed by the inclusion of neurocritical-care-specific information in a reference document, along with a periodic review of the IHT-related AEs, performing a root cause analysis as well as iteratively revising policies would only sustain quality care provided to the most vulnerable of the patients in a hospital. We proposed a 10-point template for the safe conduct of neurocritical care IHT, as shown in Table 4. 

## 5. Limitations

This study has some limitations: The limitations of survey-based research include recall and reporting bias, and there may be over-/under-reporting of adverse outcomes/events. The responses gathered reflect reported perceptions, and future studies are needed to assess the impact on patient outcomes. Overall, we had an under-representation of nursing respondents. Since nurses play an essential role in critical care IHT, we acknowledge that the survey responses by physicians may lack the perspectives and experiences that nurses may have. The lack of emphasis on neurocritical care in the guidelines makes it hard to benchmark critical aspects for neurocritical care patients against guidelines. The study’s strengths are that this was a comprehensive review of critical care IHT-related practices. This is a first step with which to explore and highlight gaps in practice as well as practice variations and identify areas for future research, in addition to those that need clarification via guidelines. We received a response rate of >60%, substantially higher than the industry standard for an external survey. We also attempted to include critical care providers with more than five years of institutional presence to present institutional knowledge of IHT practices in order to broadly reflect institutional insights. 

## 6. Conclusions

Developing a neurocritical-care-specific IHT SOP/HP and aligning practices related to the IHT of neurocritically ill patients is an urgent unmet need. Raising awareness, systematically reviewing IHT-related adverse events, and allocating IHT resources may allow for maintaining quality and safety during critical care. The lack of standardization and broad consensus-based guidelines addressing specific concerns of neurocritically ill patients are unmet needs. Variation in practices regarding neurophysiological monitoring during IHT requires in-depth inspection at the hospital level.

## Figures and Tables

**Table 1:** Safe-Neuro-Transport Study Participant and Hospital Characteristics.

		High-Income Hospital	Low–Middle-Income Hospital
	*n* = 246	*n* = 103	*n* = 143
Survey Participant Type			
Physicians not in a leadership role	107 (43.5%)	43 (41.7%)	64 (44.8%)
Nurse not in a leadership role	37 (15%)	30 (20.4%)	7 (3.8%)
Physician in a leadership role (Medical Director)	73 (29.7%)	25 (24.3%)	48 (33.6%)
Physician in a leadership role (Program Director)	26 (8.4%)	10 (9.7%)	16 (11.9%)
Nurse in a leadership role (ICU Nurse Manager)	11 (4.5%)	6 (5.8%)	5 (3.5%)
Advanced Practice Providers	9 (3.1%)	4 (3.8%)	5 (3.5%)
Experience at Current Hospital			
≥5 years	175 (71.1%)	74 (42.3%)	101 (57.7%)
<5 years	71 (28.9%)	37 (25.2%)	24 (30%)
Hospital Type			
Teaching hospital	227 (92.3%)	99 (43.6%)	128 (56.3%)
Comprehensive stroke center	153 (62.2%)	90 (58.8%)	63 (41.2%)
Level I trauma center	94 (38.2%)	61 (64.9%)	33 (35.1%)
Intensive care unit			
Hospitals with more than one	197 (80.1%)	85 (48.2%)	102 (51.8%)
Dedicated neurocritical care unit	156 (63.4%)	84 (53.9%)	72 (46.2%)
Presence of IHT SOP/HP	90 (36.6%)	44 (42.7%)	46 (32.2%)

Abbreviations: NCC: neurocritical care physician; ICU: intensive care unit; NCCU: dedicated neurocritical care unit; CC: critical care; ED: emergency medicine department; IHT: intrahospital transport; SOP: standard operating procedures; and HP: hospital policy.

**Table 2:** Variation in the preparation/assessment of critically ill patients undergoing intrahospital transport stratified by country income level.

	Overall (*n* = 246)	HIC (*n* = 103)	LMIC (*n* = 143)	Variation between Country Income Region ICC (95% CI) *	Variation between Country ICC (95% CI) *
Vasoactive medication use	70.60%	67.40%	73.80%	0 (0.00–0.02)	0.09 (0.00–0.27)
Hemodynamic data	69.85%	65.90%	73.80%	0 (0.00–0.02)	0.07 (0.00–0.24)
Examine equipment necessary	63.40%	60.50%	66.30%	0 (0.00–0.02)	0.02 (0.00–0.12)
Inspired oxygen setting	62.90%	55.80%	70%	0 (0.00–0.02)	0.11 (0.00–0.31)
Destination area ready to receive	61.05%	55.80%	66.30%	0 (0.00–0.01)	0.06 (0.00–0.20)
Identify qualified personnel	60.95%	54.40%	67.50%	0 (0.00–0.02)	0.06 (0.00–0.22)
Positive end-expiratory pressure settings	60.15%	56.50%	63.80%	0 (0.00–0.01)	0.08 (0.00–0.25)
Pulse oximetry	59%	51%	67.50%	0 (0.00–0.02)	0.06 (0.00–0.23)
Transport tolerance/sedation plans	59%	53%	65%	0 (0.00–0.02)	0.13 (0.00–0.34)
Identify overall risks associated with transport	54.45%	47.60%	61.30%	0 (0.00–0.02)	0.18 (0.00–0.38)
Interdisciplinary communication	53.65%	43.50%	63.80%	0 (0.00–0.02)	0.10 (0.00–0.28)
Chest tube status	52.80%	45.60%	60%	0 (0.00–0.02)	0.06 (0.00–0.22)
Risk stratification (low-/moderate-/high-risk)	52.25%	43.50%	61%	0 (0.00–0.02)	0.11 (0.00–0.32)
Notify ancillary services of IHT timing	52.05%	42.80%	61.30%	0 (0.00–0.014)	0.11 (0.00–0.29)
Ventilator settings	49.85%	42.20%	57.50%	0 (0.00–0.02)	0.16 (0.00–0.35)
Notify the physician accompanying patient	44.20%	29.90%	58.50%	0.02 (0.00–0.08)	0.09 (0.00–0.26)
Baseline intracranial pressure	43.20%	55.10%	31.30%	0.08 (0.00–0.19)	0.18 (0.00–0.41)
Peak airway pressure	43%	34%	51.30%	0 (0.00–0.02)	0.11 (0.00–0.31)
PaO_2_:FiO_2_ ratio	42.55%	38.80%	46.30%	0 (0.00–0.02)	0.03 (0.00–0.15)
Head of bed tolerance for intracranial/cerebral perfusion pressure	41.95%	47.60%	36.30%	0.09 (0.00–0.22)	0.10 (0.00–0.29)
Indication for IHT	40.55%	28.60%	52.50%	0 (0.00–0.02)	0.17 (0.00–0.40)
External ventricular drain clamping tolerance	39.50%	41.50%	37.50%	0.08 (0.00–0.19)	0.22 (0.00–0.46)
Neurological status documentation	39.05%	30.60%	47.50%	0 (0.00–0.01)	0.14 (0.00–0.35)
Nutrition (NPO status tube feeds)	38.00%	27.20%	48.80%	0 (0.00–0.03)	0.07 (0.00–0.24)
Neurophysiological monitoring	36.30%	38.80%	33.80%	0.07 (0.00–0.16)	0.12 (0.00–0.32)
Minute ventilation status	34.85%	27.20%	42.50%	0 (0.00–0.02)	0.14 (0.00–0.36)
Tracheostomy <7 days	32.10%	20.40%	43.80%	0.03 (0.00–0.09)	0.08 (0.00–0.27)
Baseline cerebral perfusion pressure	29.05%	38.10%	20%	0.04 (0.00–0.11)	0.13 (0.00–0.33)
Brain tissue oxygen monitoring	10.95%	15.60%	6.30%	0.08 (0.00–0.22)	0.23 (0.00–0.51)

Abbreviations: ICC: intraclass correlation coefficient; CI: confidence interval. Notes: * Because the random intercept model did not estimate variance components from sample data, the mean ICC and percentile 95% confidence interval (CI) from 1000 bootstrap samples are reported. Interpretation: The ICC reflects the variability in outcomes within and between country income regions or countries. 1. Values close to 1 imply that clustering within country incomes or countries is similar. 2. Values close to 0 imply that clustering within country incomes or countries differs.

**Table 3:** Variation in reported practices regarding equipment and monitoring standards during the intrahospital transport of neurocritically ill patients.

	Overall	HIC (*n* = 103)	LMIC (*n* = 143)	Variation between Country Income Region ICC (95% CI) *	Variation between Country ICC (95% CI) *
Pulse oximetry	93.80%	89.40%	98.20%	0 (0.00–0.01)	0.14 (0.00–0.35)
Ample oxygen source	89.95%	87.20%	92.70%	0.01 (0.00–0.05)	0.21 (0.00–0.35)
Resuscitation bag	80.50%	75.50%	85.50%	0 (0.00–0.01)	0.17 (0.00–0.40)
Additional drugs	70.50%	62.80%	78.20%	0 (0.00–0.02)	0.09 (0.00–0.27)
Resuscitation drugs	69.80%	54.20%	85.40%	0.01 (0.00–0.04)	0.19 (0.00–0.41)
Transport ventilator	68.60%	68.10%	69.10%	0.01 (0.00–0.05)	0.18 (0.00–0.39)
Stethoscope	68.55%	55.30%	81.80%	0.02 (0.00–0.059)	0.18 (0.00–0.41)
Cardiac telemonitoring	66.85%	75.50%	58.20%	0.09 (0.00–0.24)	0.20 (0.00–0.04)
Endotracheal intubation equipment	59.65%	44.70%	74.60%	0.03 (0.00–0.09)	0.30 (0.04–0.56)
Manual bag-ventilated	47.50%	27.70%	67.30%	0.04 (0.00–0.11)	0.17 (0.00–0.42)
Telephone number/pager	45.45%	30.90%	60%	0 (0.00–0.02)	0.20 (0.00–0.43)
Transport trolley	42.30%	26.60%	58%	0.03 (0.00–0.08)	0.25 (0.00–0.05)
Intensive care unit ventilator	38.40%	27.70%	49.10%	0 (0.00–0.02)	0.23 (0.00–0.05)
Defibrillator	35.50%	38.30%	32.70%	0.02 (0.00–0.09)	0.16 (0.00–0.38)
Elevator key	34.55%	30.90%	38.20%	0 (0.00–0.02)	0.17 (0.00–0.39)
Intracranial pressure	32.85%	45.70%	20%	0.06 (0.00–0.18)	0.28 (0.00–0.55)
Portable suction	29.80%	28.70%	30.90%	0 (0.00–0.02)	0.15 (0.00–0.38)
End-tidal carbon dioxide	28.60%	24.50%	32.70%	0 (0.00–0.02)	0.46 (0.07–0.71)
Brain tissue oxygenation	8.60%	11.70%	5.50%	Not calculated due to low overall prevalence	Not calculated due to low overall prevalence

Abbreviations: HIC: high-income country; LMIC: low–middle-income country. Notes: ICC: intraclass correlation coefficient; CI: confidence interval. Notes: * Because the random intercept model did not estimate variance components from sample data, the mean ICC and percentile 95% confidence interval (CI) from 1000 bootstrap samples are reported. Interpretation: The ICC reflects the variability in outcomes within and between country income regions or countries. 1. Values close to 1 imply that clustering within country income or countries is similar. 2. Values close to 0 imply that clustering within country income or countries differs.

**Table 4:** Proposed neurocritical-care-specific IHT policy and procedure template.

1. Scope of the policy	Adults/children Applies to all ICUs vs. specific (for example, a neurocritical care unit)
2. Purpose of the policy	Quality and patient safety
3. Purpose of transport	Routine, urgent, and emergent Diagnostic vs. therapeutic/interventional
4. Risk stratification of patients	Low-, moderate-, or high-risk Intolerance to tolerate head-of-bed of zero degrees Intolerance to external ventricular and lumbar drain clamping Indication for sedation/analgesia for the procedure Underlying pathology
5. Peri-transport communication	Confirmation with the neurocritical care provider of the urgency of IHT Communication of the need for physician or advanced practice provider presence Communication with the receiving team
6. Pre- and post-transport examination as well as documentation	Neurological exam, including the Glasgow Coma Scale (GCS) score Intracranial pressure and cerebral perfusion pressure, brain-tissue oxygenation External ventricular and lumbar drain settings, cerebrospinal fluid output External ventricular and lumbar drain clamp tolerance Blood pressure targets PaO_2_, PCO_2_ targets Notification of respiratory therapists Debriefing points in the event of AEs
7. Transport equipment, safety, and monitoring	Neurological: Intracranial pressure, cerebral perfusion pressure, brain tissue oxygenation, and end-tidal carbon dioxide Dedicated intravenous pole to mount external ventricular and lumbar drain-related CSF collecting systems Vasoactive agents to maintain cerebral perfusion pressure Osmolar agents (mannitol/hypertonic saline) to manage intracranial pressure crises
8. Quality assurance review	Reporting of adverse events to the QI database Recurrent meetings Multidisciplinary quality review
9. Revisions	Multidisciplinary group Iterative process
10. References	Guidelines Recommendations

## Data Availability

The data collected are unavailable to be shared publicly.

## Supplementary Materials

The following supporting information can be downloaded at: https://www.mdpi.com/article/10.3390/jcm12093183/s1, File S1: Membership of the Safe-Neuro-Transport Collaborators.
