# Supplementary material for: Benchmarking Hospital Practices and Policies on Intrahospital Neurocritical Care Transport: The Safe-Neuro-Transport Study
Source: J Clin Med. 2023 Apr 28;12(9):3183. doi: 10.3390/jcm12093183 (PMC10179223; doi:10.3390/jcm12093183)
Supplement: Supplementary file 1 [file jcm-12-03183-s001.zip › jcm-2318372-supplementary.docx]

Safe-Neuro-Transport Collaborators

| **Country** | **Name of Collaborator** |
| --- | --- |
| Argentina | Eleonora Roxana Cunto, Javier Pérez Flores, Santiago Manuel Bondone, Marino Maria Rosa, Yohana Quiroga, Walter Videtta, Paredes Walter Horacio, Gabriela Aparicio, Milton Villalobos, Nicolás Sebastián Rocchetti, Leandro Tumino, Gustavo Domeniconi, Ana María Mazzola, Francisco Javier Sotomayor, Iona Mónica Garcia |
| Australia | Deepak Bhonagiri |
| Brazil | Miren Bengoetxea Ibarrondo, Wellingson Silva Paiva |
| Chile | María Alejandra Faundez Torres, Sergio Aguilera Rodríguez, Luis Castillo F, |
| Colombia | Juan Diego Ciro, Jorge H. Mejia-Mantilla |
| Cuba | Caridad de Dios Soler Morejón |
| Nepal | Prasanna Karki, Ritesh Lamsal, Gentle Sunder Shrestha |
| Ecuador | Morocho Tutillo Diego Rolando, Alexandra Matilde Saraguro Orozco |
| Finland | Ari J Katila |
| Germany | Moritz Stuplich, Matthias Wittstock, Patrick Czorlich |
| India | S.B. Mohapatra, Sanghamitra Mishra, Kallol Deb, Subhal Dixit, Zulfiqar Ali, Neeta Vikram Karmarkar, Kuldeep Dalal, Ms.Venda,Mr.Justin, Prashant Kumar, Mayank Kumar Tyagi, Devendra Gupta, Vanitha Rajagopalan, Sridhar Nagaiyan, Shalini Nair, Yash Javeri, Kavita Sandhu, S. Manikandan |
| Italy | Alessandro De Cassai, Raffaele Aspide, Chiara Robba |
| Mexico | Julio César Mijangos-Méndez, Juvenal Franco Granillo, Maricela García Arellano |
| Paraguay | Julio Apodaca Guex, Natalia Gomez Arriola |
| Peru | Maria Mercedes Chumbe Mendoza |
| Philippines | Gemmalynn Burahan Sarapuddin |
| Portugal | Celeste Dias |
| Spain | Susana Altaba Tena, Bárbara Vidal Tegedor |
| Sri Lanka | Shanmugam Puvanendiran, William Gopallawa Mawata |
| Spain | Funes Nelson Nydia |
| Thailand | Jirapong Vongsfak, Chalermwoot Puttima, Thitikan Wangapakul, Poonyanuch Charoenkoop, Suttasinee Petsakul |
| UAE | Yasser B. Abulhasan, Hosam M. Al-Jehani, Naeema Ali |
| USA | Christa O'Hana S. Nobleza, Swarna Rajagopalan, Lakeshia Moten, Jamey Hammock, Amay Parikh, Aarti Sarwal, Angela Hays Shapshak, Yama Akbari, Sarah Bockian, Andrea Blanchette, Meg Caiazzo, Audrey Paulson, Gabriel V. Fontaine, Ivan Da Silva, Amanda Pilecki, Stephanie Smith, Dawn Meadows, Marget Smallwood, Michele Wilkerson, Anna M. Cervantes-Arslanian, Keith Dombrowski, Jennifer R. Glover, David A. Wyler, Craig A. Williamson, Wanda J. Handel, Debra E Roberts, Julius Gene Latorre |
| Ukraine | Sergii Gorbachov |
| Venezuela | Jacobo Mora |
